# Improving long COVID-related text classification: a novel end-to-end domain-adaptive paraphrasing framework

**DOI:** 10.1038/s41598-023-48594-4

**Published:** 2024-01-02

**Authors:** Sai Ashish Somayajula, Onkar Litake, Youwei Liang, Ramtin Hosseini, Shamim Nemati, David O. Wilson, Robert N. Weinreb, Atul Malhotra, Pengtao Xie

**Affiliations:** 1 Department of Electrical and Computer Engineering, University of California, La Jolla, San Diego, USA; 2 Division of Biomedical Informatics, University of California, La Jolla, San Diego, USA; 3 Department of Medicine, University of Pittsburgh Medical Center, Pittsburgh, USA; 4 Hamilton Glaucoma Center, Shiley Eye Center and Department of Ophthalmology, University of California, La Jolla, San Diego, USA; 5 UC San Diego Health, Department of Medicine, La Jolla, San Diego, USA

**Keywords:** Viral infection, Epidemiology, Machine learning

## Abstract

The emergence of long COVID during the ongoing COVID-19 pandemic has presented considerable challenges for healthcare professionals and researchers. The task of identifying relevant literature is particularly daunting due to the rapidly evolving scientific landscape, inconsistent definitions, and a lack of standardized nomenclature. This paper proposes a novel solution to this challenge by employing machine learning techniques to classify long COVID literature. However, the scarcity of annotated data for machine learning poses a significant obstacle. To overcome this, we introduce a strategy called medical paraphrasing, which diversifies the training data while maintaining the original content. Additionally, we propose a Data-Reweighting-Based Multi-Level Optimization Framework for Domain Adaptive Paraphrasing, supported by a Meta-Weight-Network (MWN). This innovative approach incorporates feedback from the downstream text classification model to influence the training of the paraphrasing model. During the training process, the framework assigns higher weights to the training examples that contribute more effectively to the downstream task of long COVID text classification. Our findings demonstrate that this method substantially improves the accuracy and efficiency of long COVID literature classification, offering a valuable tool for physicians and researchers navigating this complex and ever-evolving field.

## Introduction

The COVID-19 pandemic, caused by the SARS-CoV-2 virus, has left an indelible mark on global health, infecting over 763 million people and resulting in more than 6.9 million deaths^1–3^. Through the relentless efforts of healthcare professionals and researchers worldwide, the severity of the pandemic has been mitigated. However, a notable proportion of COVID-19 patients persistently report residual symptoms and health complications following the resolution of the acute phase of the disease^4,5^. This persistent manifestation has led to the identification of a complex and puzzling condition known as “Post-Acute Sequelae of SARS-CoV-2 infection” (PASC) or long COVID^6^. Current research and epidemiological surveys indicate that between 10% and 30% of COVID-19 survivors may experience these protracted symptoms^7–9^. Characterized by multisystemic manifestations such as respiratory complications, cardiovascular disorders, cognitive impairments, and severe fatigue^10–13^, long COVID often persists for numerous months post-infection. As such, a thorough understanding of the pathophysiology and long-term consequences^14–16^ of long COVID is of paramount importance to inform strategies for its management and prevention.

To remain abreast of the evolving nature of long COVID, physicians and researchers frequently resort to the extensive array of research articles and related works. However, a significant hurdle in advancing the understanding of long COVID and in developing efficacious management strategies is the daunting task of identifying pertinent articles within the broad existing literature. The scientific landscape of this novel condition is characterized by a substantial variation in the employed definitions of long COVID^17–19^ across diverse studies, creating a formidable challenge for physicians in their pursuit of finding relevant resources. This inconsistency not only complicates the task of extracting relevant long COVID articles but also engenders a dilemma in the query process. Precise searches using terms such as “post-acute sequelae of SARS-CoV-2 infection” often yield limited results, owing to the specificity of the terminology. In contrast, broader search terms like “post-COVID symptoms” can generate a plethora of results, having false positives due to the generic nature of the terminology^20^. Compounding these challenges, while consensus-based case definitions are gradually solidifying, the majority of publications tend to describe the condition without explicitly designating it as long COVID, creating inconsistencies in the literature^21^. This lack of standardized nomenclature can be attributed to the novelty of long COVID as a distinct clinical entity and the ongoing evolution of our understanding of its diverse manifestations.

In tackling this issue, we advocate for the application of machine learning specifically, text classification, to classify medical articles. Text classification, a key task in machine learning, is a technique that categorizes input sentences based on their content and has found particular utility in the medical domain. In this context, text classification has been instrumental in simplifying the categorization of complex medical literature, such as articles on Cancer Susceptibility Genes and reports in the US Vaccine Adverse Event Reporting System (VAERS)^22,23^. Text Classifiers, once trained, evaluate and categorize documents according to their inherent content. This application has effectively reduced the workload of human experts, and enabled a more efficient and organized approach to literature review. These successful implementations substantiate our motivation to employ text classification for the classification of long COVID articles, an application that could provide valuable assistance to doctors.

A significant challenge in developing high-performance machine learning models lies in the availability of ample annotated data, which is often required in the thousands. This challenge is particularly pronounced in the case of long COVID articles, where obtaining such data necessitates skilled human intervention, making it a laborious task. Several methods in the Natural Language Processing (NLP) literature have been proposed to address this data scarcity and train efficient machine learning models. Among these methods are Back Translation^24^, Synonym Replacement^25^, and EDA^26^. These techniques attempt to mitigate data scarcity by employing simple heuristic-based operations (random insertion, deletion, swapping, and synonym replacement) or leveraging another language model. However, they exhibit certain limitations. For instance, they often produce limited and simple text variations through random insertion, deletion, and so on. Additionally, some of these methods, particularly those based on language models, risk generating ‘hallucinations’-alterations that can distort the original text’s meaning or context. Techniques that diverge significantly from the target domain or context might generate less relevant training data. This could potentially undermine the classifier’s ability to generalize for specific tasks, thus affecting the overall performance of the machine learning model. Therefore, addressing these limitations is of paramount importance.

**Figure 1:**
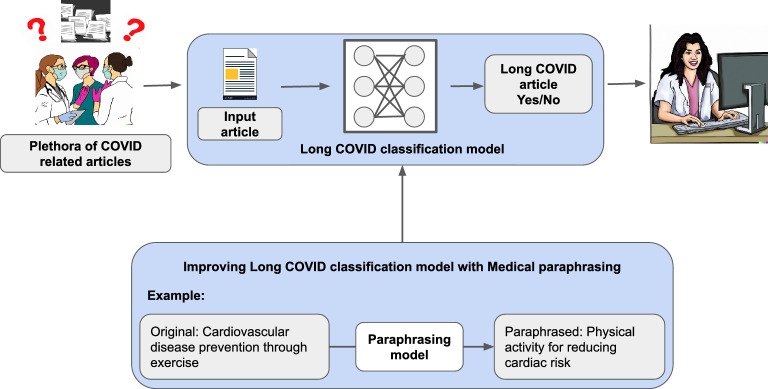
An illustration of our approach. Leveraging medical paraphrasing to overcome annotated data scarcity in training an efficient long COVID article classification model.

To address the challenge of data scarcity, we introduce the concept of medical paraphrasing. This technique generates alternative versions of training texts, maintaining the original medical context and semantics. These paraphrased texts, preserving the core intent of the original sentences, serve as an expanded dataset to alleviate data scarcity. The intuition is further explained pictorially in Fig 1. Importantly, the class label assigned to these generated paraphrases aligns with the class label of their corresponding original sentence. This approach facilitates a diverse representation of training data, enhancing the machine learning model’s capacity for generalization. We propose the use of a paraphrasing model^27^ for this task, that takes an input text and generates a paraphrased version. A key challenge is the lack of a specific paraphrasing dataset for long COVID, ideally containing long COVID-related sentences and their rephrased counterparts as training data. To circumvent this, we intend to train the paraphrasing model on a generic paraphrasing dataset, which is readily available. However, this approach has its challenges. Training a paraphrasing model on a generic dataset may yield rephrased versions that deviate from the long COVID domain or generate hallucinations, as previously discussed.

In order to address the challenges outlined, we introduce a data-reweighting-based multi-level optimization framework for domain adaptive paraphrasing. This framework is reinforced by a Meta-Weight-Network (MWN) and is designed to leverage the feedback from the long COVID text classification model to influence the training of the paraphrasing model. Our proposed Multi-Level Optimization (MLO) framework unfolds over three stages. A multi-level optimization problem is an extension of a Bi-Level Optimization problem^28^ (BLO). In the initial stage, the paraphrasing model is trained on a general domain paraphrasing dataset, using a data reweighting strategy. Every paraphrasing training example is assigned a weight, denoted by $a_i$, within the range of [0,1]. To predict these data weights, we employ a Multi-Layer-Perceptron (MLP) layer, referred to as a Meta-Weight-Network (MWN). This network operates by taking the loss associated with each pair of original and paraphrased sentences, $\{t_i, s_i\}$, as input and subsequently outputs a scalar weight, $a_i$, for the *i*-th training example pair. The MWN is designed to account for the domain difference between the paraphrasing data example and the long COVID text classification dataset. A larger $a_i$ implies higher importance of the paraphrasing training example for the long COVID text classification task, and vice versa. The second stage involves feeding the text classification training examples into the paraphrasing model to generate auxiliary data for the text classification model. In the final stage, the MWN weights are fine-tuned by minimizing the validation loss of the text classification model. This step serves as a feedback mechanism, guiding the paraphrasing model to improve its generations. Through this process, we ensure that the domain of the generations from the paraphrasing model aligns with the domain of the long COVID-related articles, optimizing the overall performance.

## Related work

### Text classification for long COVID

 A recent study^29^ endeavored to develop a classification system for long COVID to assist clinicians in providing individualized care. Using Hierarchical Ascendant Classification (HAC), the research identified three distinct symptom patterns of long COVID, suggesting a gradient in disease severity. This classification implies the potential to subdivide long COVID into three severity-based subcategories, a significant step towards personalized patient care. Parallel efforts^30^ have been made to comprehend the societal sentiment surrounding long COVID, through the classification of Twitter users’ sentiments. By analyzing social media data, researchers aimed to capture the overall emotional tone related to long COVID, thereby contributing to our understanding of the public response to this condition. The identification of individuals suffering from long COVID is crucial for delivering adequate support. To this end, an XGBoost machine learning approach^31^ was devised using the National COVID Cohort Collaborative’s (N3C) electronic health record repository. This approach aimed to pinpoint patients likely to be affected by long COVID. However, the nascent nature of long COVID and the scarcity of related data pose significant challenges. The lack of standardized or consensus terminology for long COVID^32^ complicates the identification of relevant scientific articles, thereby obstructing the construction of accurate machine-learning models that could assist medical professionals. In fact, some articles discussing long COVID do not explicitly label the condition, further complicating the task of creating effective data-driven tools for healthcare providers. Our work aims to address this issue.

### Methods to address data scarcity

 To build such applications, the problem of data scarcity must be overcome. Studies have been carried out to tackle data scarcity in NLP^33,34^. A heuristic-based technique^26^ was proposed to boost the performance of text classification tasks by randomly inserting, deleting, swapping, and replacing words in the text. Another approach was to replace a given word with a word predicted by a bi-directional language model^35^. Studies have shown that keyword replacement with hypernyms and character-level synthetic noise are effective techniques for addressing data deficiency^36^. Reinforcement learning guided conditional generation was proposed to tackle the data deficiency problem^37^. Back-translation techniques, where sentences are translated into another language and then back into the original language, have been used as auxiliary data for Machine Translation^24^. After the success of ChatGPT, a novel technique named AugGPT^38^ was proposed, breaking down each sentence in training samples into multiple conceptually related but semantically distinct samples. AugGPT shows superior performance in terms of the distribution of augmented samples and accuracy over few-shot learning text classification tasks. However, these methods may generate noisy data which might not be optimal for the medical domain because there is no feedback mechanism to ensure the generations are suitable for the downstream long COVID article classification model. Our method addresses this issue by incorporating a feedback mechanism to ensure the generations are in the medical domain and further aids the downstream long COVID article classification model.

### Bi-level optimization

 Bi-Level Optimization^28^ (BLO) is a class of optimization problems that involves solving two optimization problems, namely lower and upper optimization problems, simultaneously, with one problem nested within the other. The optimal solution to the lower optimization problem constraints the objective function of the upper optimization problem. However, the optimal parameters of the lower problem are reliant on the optimal solution of the upper problem, creating a interdependency between the two stages. A multi-level optimization problem is an extension of bi-Level optimization problem with more than problems in the lower stage. The use of bi-level optimization methods has been successfully demonstrated in various machine learning tasks. For instance, BLO^39^ has been applied to the problem of neural architecture search, configuring the neural architecture and model weights as the upper and lower parameters, respectively. Similarly, data selection problems^40–42^ have been represented as bi-level optimization problems, with the upper and lower variables being the data weights and the model weights, respectively. Further applications of bi-level optimization frameworks have been seen in hyperparameter optimization^43^, label correction^44^, training data generation^45^, data augmentation^46^, and learning rate adaptation^47^. In each of these applications, the model weights, which are the lower parameters, are optimized by minimizing the training loss, while the upper parameters such as neural architecture, hyperparameters, data weights, and so on. are learned by minimizing the validation loss.

## Method

### Overview

In this section, we present our proposed solution for addressing data scarcity in long COVID-related article classification. Our approach utilizes a paraphrasing model that incorporates feedback from the text classification model. The paraphrasing model rephrases the input training texts to generate an additional training dataset. This, in conjunction with the original training dataset, is utilized to train the long COVID article classification model. The performance of the paraphrasing model is evaluated and improved based on the validation performance of the long COVID article classification model, thereby serving as a feedback mechanism. Additionally, we describe our Meta-Weight-Network, which facilitates data reweighting in the paraphrasing model, thereby tailoring the domain to the text classification model and leveraging it as feedback.

**Table 1:** Notations used to define our framework.

Notation	Meaning
*W*	Meta-Weight-Network (MWN) parameters
*C*	Long COVID article classification model parameters
*S*	Paraphrasing model parameters
${\mathscr {D}}^{S} = \{ (t_i, s_i) \}_{i=1}^M$	Paraphrasing dataset
${\mathscr {D}}^{tr} = \{ (x^{tr}_i, y^{tr}_i) \}_{i=1}^{N}$	Long COVID Training dataset
${\mathscr {D}}^{val} = \{ (x^{val}_i, y^{val}_i) \}_{i=1}^{N}$	Long COVID Validation dataset
${\mathscr {D}}^{\text {MLO-tr}}$	MLO Training dataset, 80% split of ${\mathscr {D}}^{tr}$
${\mathscr {D}}^{\text {MLO-val}}$	MLO Validation dataset, remaining 20% split of ${\mathscr {D}}^{tr}$
${\mathscr {G}}(\cdot ,S)$	Additional training dataset generated by the paraphrasing model *S*.

We summarize our notations in Table 1. The paraphrasing model is trained on a paraphrasing dataset ${\mathscr {D}}^{S}$, where the model takes an article as input and learns to output its paraphrased version. A long COVID text classification model is trained on a training dataset, ${\mathscr {D}}^{tr}$ and validated on a validation dataset, ${\mathscr {D}}^{val}$, where we have labels indicating whether an article is long COVID related. To facilitate the training of our MLO framework, we randomly split ${\mathscr {D}}^{tr}$ into two sets, an 80% portion for MLO-train (${\mathscr {D}}^{\text{MLO-tr}}$) and a 20% portion for MLO-validation (${\mathscr {D}}^{\text{MLO-val}}$). These sets are used for multi-level optimization, which will be discussed further in subsequent sections.

Since the long COVID-related training dataset ${\mathscr {D}}^{tr}$ is limited in size, we generate an additional dataset for training the long COVID text classification model. Specifically, we paraphrase the training examples, by passing a training text example $x^{\text {MLO-tr}}_i$ into the paraphrasing model, which outputs ${\hat{x}}^{\text {MLO-tr}}_i$. The original labels are associated with these generated texts since we assume that the paraphrasing operation does not alter the label category. Therefore, (${\hat{x}}^{\text {MLO-tr}}_i, y^{\text {MLO-tr}}_i$) is the generated pair of the original pair ($x^{\text {MLO-tr}}_i, y^{\text {MLO-tr}}_i$).

**Figure 2:**
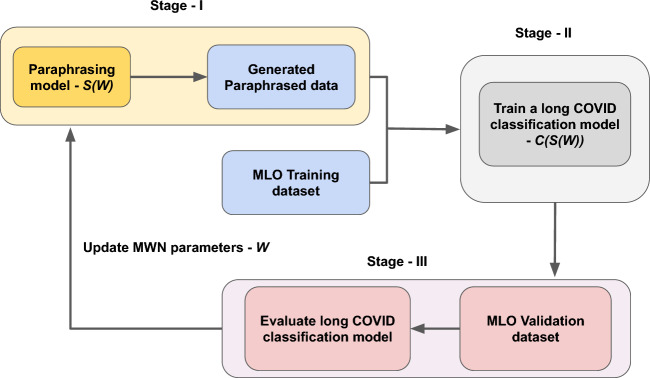
Our end-to-end data-reweighting-based multi-level optimization framework for domain adaptive paraphrasing. MWN refers to the meta-weight-network for data reweighting.

Our end-to-end framework is composed of three stages. The first stage involves training a paraphrasing model on ${\mathscr {D}}^{S}$, with each training example being weighted by a data weight output from the meta-weight network to account for the domain difference between the paraphrasing and text classification datasets. In the second stage, the text classification model is trained on ${\mathscr {D}}^{\text {MLO-tr}}$ along with the generations from the paraphrasing model trained in the first stage. In the third stage, we learn the meta-weight-network parameters by minimizing the MLO validation loss of the text classification dataset. The text classification model is evaluated on the MLO-validation dataset, ${\mathscr {D}}^{\text {MLO-val}}$, and the meta-weight-network parameters are learned by minimizing this MLO-validation loss. This approach enforces a mutual dependency between the data generation process and the text classification dataset, allowing them to interact and benefit from each other in an end-to-end fashion. We provide a detailed explanation of each section in the following sections. Fig. 2 illustrates the overall framework pictorially.

### Stage I

In this stage, we train a BART^48^ based paraphrasing model denoted by *S*. It is an encoder-decoder-based pretrained transformer model. It is trained on ${\mathscr {D}}^{S}$, which contains pairs of sentences or phrases that have the same meaning but are phrased differently. Creating a paraphrasing dataset in the medical domain is challenging due to limited resources, privacy concerns, and the need for expert involvement. Therefore, a publicly available non-medical text paraphrasing dataset is used to ensure the method is generic and does not require additional dataset creation. However, to ensure the generations are in the medical domain, data reweighting is proposed to account for the domain discrepancy. Each training example of the paraphrasing model is associated with a data weight $a_i \in [0,1]$ to account for the domain discrepancy with the downstream text classification dataset. If a paraphrasing training example deviates hugely from the domain of the text classification dataset, then the associated data weight $a_i \approx 0$ and vice-versa.

We introduce the Meta-Weight-Network (MWN) as a means of predicting the data weights. The MWN, denoted by *W*, is a multi-layer perceptron network used to approximately estimate the data weights distribution^49^. For each training example $\{(t_i, s_i)\}$, we input the associated loss into the MWN, which outputs a scalar value $a_i$ representing the corresponding data weight. The following optimization problem is solved in this stage:

1 $$\begin{aligned} S^{*}(W) = \min _{S} \sum \limits _{i=1}^M W(l(S, t_{i}, s_{i})) \cdot l(S, t_{i}, s_{i}) \end{aligned}$$

where *W* is MWN and $l(\cdot )$ is the teacher-forcing loss. The loss of each training example $(t_i,s_i)$ is weighted by its corresponding data weight $a_i$. The data weight $a_i$ is associated with each $(t_i, s_i)$ pair, and if a pair deviates significantly from the text classification dataset, its associated $a_i$ must be close to 0. The optimal paraphrasing model weights $S^*$ depend on *W*, as the loss function in Eq. (1) is dependent on *W*. The parameters of *W* are not learned in this stage; otherwise, *W* weights will be learned such that all the $a_i$ become 0, which is a degenerate solution. Instead, *W* weights are updated in a later stage.

### Stage II

In the second stage, we generate the auxiliary dataset using the paraphrasing model trained above $S^*(W)$ and further use it to train the text classification model, denoted by *C*. The MLO-train dataset ${\mathscr {D}}^{\text {MLO-tr}}$ is used to train the text classification dataset. Given a training example pair $(x^\text {{MLO-tr}}_i, y^\text {{MLO-tr}}_i)$, the input text $x^\text {{MLO-tr}}_i$ is passed through $S^*(W)$ to generate its corresponding text ${\hat{x}}^\text {{MLO-tr}}_i$. As explained above, the label is preserved as the original label. Thus, $({\hat{x}}^\text {{MLO-tr}}_i, y^\text {{MLO-tr}}_i)$ is treated as the augmentation of $(x^\text {{MLO-tr}}_i, y^\text {{MLO-tr}}_i)$. This process is repeated for all the training examples in ${\mathscr {D}}^{\text {MLO-tr}}$ to generate additional dataset ${\mathscr {G}}({\mathscr {D}}^{\text {MLO-tr}},S^{*}(W))$. Given this generated dataset, *C* is trained on ${\mathscr {D}}^{\text {MLO-tr}}$ and ${\mathscr {G}}({\mathscr {D}}^{\text {MLO-tr}},S^{*}(W))$. The following optimization problem is solved in the second stage:

2 $$\begin{aligned} \begin{array}{l} C^{*}(S^{*}(W)) = {\text {min}_{C}} L(C, {\mathscr {D}}^{\text {MLO-tr}}) + \gamma L(C, {\mathscr {G}}({\mathscr {D}}^{\text {MLO-tr}},S^{*}(W))) \end{array} \end{aligned}$$

where $\gamma$ is a tradeoff parameter and $L(\cdot )$ denotes a cross-entropy classification loss. $L(C, {\mathscr {D}}^{\text {MLO-tr}})$ denotes the loss defined on the MLO-train dataset and $L(C, {\mathscr {G}}({\mathscr {D}}^{\text {MLO-tr}},S^{*}(W)))$ is the loss defined on the generated dataset. The trade-off parameter $\gamma$ controls the contribution of the loss associated with the generated dataset. The optimal classification model weights depend on $S^*(W)$ from the second term in Eq. (2), the loss associated with the generated dataset. This term depends on $S^*(W)$, which generates the auxiliary dataset.

### Stage III

In this stage, the W parameters are learned by minimizing the MLO-validation loss of the text classification model, which is the loss of the text classification model evaluated on ${\mathscr {D}}^{\text {MLO-val}}$ dataset. These learned W weights thus influence the predicted data weights $a_i's$, which tailor to the domain of the text classification model, thereby acting as a feedback loop.

3 $$\begin{aligned} \min _{W} \quad L(C^{*}(S^{*}(W)), {\mathscr {D}}^{\text {MLO-val}}) \end{aligned}$$

### A three-level optimization framework

We unify the above three stages into a multi-level optimization framework as follows.

4 $$\begin{aligned} \begin{array}{ll} {\text {min}_{W}}\quad &{} L(C^{*}(S^{*}(W)), {\mathscr {D}}^{\text {MLO-val}})\\ \text {s.t.} \quad &{} C^{*}(S^{*}(W)) = {\text {min}_{C}}L(C, {\mathscr {D}}^{\text {MLO-tr}}) + \gamma L(C, {\mathscr {G}}({\mathscr {D}}^{\text {MLO-tr}},S^{*}(W))) \\ &{} S^{*}(W) = {\text {min}_{S}}\sum \limits _{i=1}^M W(l(S, t_{i}, s_{i})) \cdot l(S, t_{i}, s_{i}) \end{array} \end{aligned}$$

The three stages defined above are performed end-to-end with interleaved dependency. The solution of stage I, $S^*(W)$, is used in stage II to generate data. The generated data, along with original training data, is used to train the classification model, the obtained solution is $C^*(S^*(W))$, which is then evaluated on ${\mathscr {D}}^{\text {MLO-val}}$ in stage III. The Meta-Weight-Network parameters (W) are learned by minimizing this validation loss which acts as feedback in stage III. The solution learned in this stage ($W^\prime$) influence the solution of stage II, $C^*(S^*(W^\prime ))$, and thereby changing solution obtained in stage I, $S^*(W^\prime )$.

### Optimization algorithm

**Algorithm 1:**
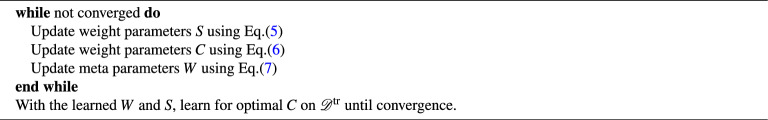
Optimization algorithm

This section uses a gradient-based optimization algorithm to solve the MLO problem in Eq. (4). One step gradient descent^39^ of *S* is used to approximate $S^{*}(W)$:

5 $$\begin{aligned} \begin{aligned} \quad&S^{*}(W) \approx S^{\prime } = S - \eta _{s}\nabla _{S} \left( \sum \limits _{i=1}^M W(l(S, t_{i}, s_{i})) \cdot l(S, t_{i}, s_{i}) \right) \end{aligned} \end{aligned}$$

We substitute $S^{*}(W) \approx S^{\prime }$ into the next level objective function to solve for the optimal text classification model parameters. $C^{*}(S^{*}(A))$ is approximated using one-step gradient descent of *C*:

6 $$\begin{aligned} \begin{array}{ll} \quad&C^{*}(A) \approx C^{\prime } = C - \eta _{c} \left( \nabla _{C} (L(C, {\mathscr {D}}^{\text {MLO-tr}}) + \gamma L(C, {\mathscr {G}}({\mathscr {D}}^{\text {MLO-tr}},S^{\prime }))) \right) \end{array} \end{aligned}$$

The optimal W parameters are learned by gradient descent of the objective function of stage III, in which the above obtained $C^{*}(A) \approx C^{\prime }$ is substituted.

7 $$\begin{aligned} \begin{aligned} \quad&W \leftarrow W - \eta _{w}\nabla _{W} L(C^{\prime }, {\mathscr {D}}^{\text {MLO-val}}) \end{aligned} \end{aligned}$$

where

8 $$\begin{aligned} \begin{array}{ll} \nabla _{W} L(C^{\prime }, {\mathscr {D}}^{\text {MLO-val}}) = \frac{\partial S^{\prime }}{\partial W}\frac{\partial C^{\prime }}{\partial S^{\prime }}\frac{\partial L(C^{\prime }, {\mathscr {D}}^{\text {MLO-val}})}{\partial C^{\prime }} =\\ \eta _s\eta _c\gamma {\nabla }^{2}_{W,S} \left( \sum \limits _{i=1}^M W(l(S, t_{i}, s_{i})) \cdot l(S, t_{i}, s_{i})\right) {\nabla }^{2}_{S^{\prime },C}L(C, {\mathscr {G}}({\mathscr {D}}^{\text {MLO-tr}},S^{\prime })){\nabla }_{C^{\prime }}L(C^{\prime }, {\mathscr {D}}^{\text {MLO-val}}) \end{array} \end{aligned}$$

Finite difference approximation reduces the computational complexity of expensive matrix-vector products in Eq. (8).

9 $$\begin{aligned} \begin{array}{ll} \approx \frac{\eta _s\eta _c\gamma }{2\alpha }\{[{\nabla }_{S^{\prime }}L(C^{+}, {\mathscr {G}}({\mathscr {D}}^{\text {MLO-tr}},S^{\prime })) - {\nabla }_{S^{\prime }}L(C^{-}, {\mathscr {G}}({\mathscr {D}}^{\text {MLO-tr}},S^{\prime }))] {\nabla }^{2}_{W,S}\sum \limits _{i=1}^M W(l(S, t_{i}, s_{i})) \cdot l(S, t_{i}, s_{i})\} \end{array} \end{aligned}$$

where,

$$\begin{aligned} \alpha = \frac{0.01}{\left\Vert {\nabla }_{C^{\prime }}L(C^{\prime }, {\mathscr {D}}^{\text {MLO-val}})\right\Vert _{2}}, C^{\pm } = C \pm \alpha {\nabla }_{C^{\prime }}L(C^{\prime }, {\mathscr {D}}^{\text {MLO-val}}). \end{aligned}$$

Eq. (9) can be further approximated by:

10 $$\begin{aligned} \begin{array}{ll} \frac{1}{\alpha ^{\pm }_{S}} \{{\nabla }_{W}\sum \limits _{i=1}^M W(l(S^{+}_{\pm }, t_{i}, s_{i})) \cdot l(S^{+}_{\pm }, t_{i}, s_{i}) - {\nabla }_{W}\sum \limits _{i=1}^M W(l(S^{-}_{\pm }, t_{i}, s_{i})) \cdot l(S^{-}_{\pm }, t_{i}, s_{i})\} \end{array} \end{aligned}$$

where, $$\alpha ^{\pm }_{S} = \frac{0.01}{\left\Vert {\nabla }_{S^{\prime }}L(C^{\pm }, {\mathscr {G}}({\mathscr {D}}^{\text {MLO-tr}},S^{\prime }))\right\Vert _{2} }$$, $$S^{\pm }_{+} = S \pm \alpha ^{+}_{S}{\nabla }_{S^{\prime }}L(C^{+}, {\mathscr {G}}({\mathscr {D}}^{\text {MLO-tr}},S^{\prime }))$$, $$S^{\pm }_{-} = S \pm \alpha ^{-}_{S}{\nabla }_{S^{\prime }} L(C^{-}, {\mathscr {G}}({\mathscr {D}}^{\text {MLO-tr}},S^{\prime }))$$

We perform these update steps alternatively until convergence. Then the classification model is further trained on the entire training dataset ${\mathscr {D}}^{tr}$ for a few iterations until convergence. The overall algorithm is presented in Algorithm 1.

## Experiments

### Dataset

The dataset used in this work for long COVID-related article classification is publicly available on HuggingFace^50^. Given the difficulty in obtaining annotated long COVID-related article datasets due to the previously discussed challenges, we conduct comprehensive experiments on this readily available dataset. The dataset was manually curated by domain experts, with the initial subset gathered by experts from the Robert Koch Institute (RKI). They collected the data by querying a variety of related search strings in the PubMed database^51^ and other COVID-related databases. Additionally, data was sourced from the ‘long COVID research library’ released by Pandemic-Aid Networks, which has compiled crucial articles on long COVID. The dataset is binary and classifies documents into two categories: non-long COVID (labeled by 0) and long COVID (labeled by 1) related documents. The text of this dataset predominantly comprises titles and abstracts that succinctly summarize the research articles. The distribution of the dataset is as follows: the training set consists of 207 examples, the validation set contains 207 examples, and the test set includes 138 examples. For training the paraphrasing model, we employ the MRPC dataset^52^, which is specifically curated for paraphrasing tasks. In this study, a subset of approximately 500 training examples from this dataset is utilized to finetune the paraphrasing model.

### Baselines

We compare our method with the following baselines. **Vanilla**: Vanilla training of the text classifier on the given training dataset. **EDA**^26^: EDA is a heuristic-based technique to address data deficiency. The authors propose the following operations: random insertion, synonym replacement, random swap, and random deletion. **Back translation**^24^: Back translation is performed by translating texts from the source language to a target language using a trained language model. Then these texts in the target language are translated back into the source language using another trained language model to be used as auxiliary data. We use a pretrained Opus-MT-based^53^ sequence-to-sequence model pretrained on the English-French language for back translation. **T5 abstractive summarization**: To address the issue of data scarcity, text summarization is conducted on input texts, generating concise summaries by employing the T5-large model^54^. **Keyword replacement**: We adopted a keyword replacement strategy using a comprehensive list of terms related to long COVID, as detailed in^50^. The keywords utilized are PASC, long COVID, long term COVID effects, post-acute sequelae, post-acute sequelae of SARS-CoV-2, long-haul COVID, post-acute COVID syndrome, persistent COVID-19, post-acute COVID19 syndrome, long hauler COVID, longCOVID, post-acute sequelae of SARS-CoV-2 infection, long haul COVID, chronic COVID syndrome, and long-COVID. In our approach, we generated 16 unique augmentations for each input text by replacing each occurrence of a keyword with another term from the aforementioned list, contingent on the keyword’s presence in the text, following EDA^26^.

### Experimental setup

The model used for text classification in EDA^26^ has been used in this work. The model consists of an input layer followed by 64 hidden LSTM units (bidirectional layer), a dropout layer with a probability of 0.5, a bi-directional layer with 32 LSTM units, and another dropout layer with a probability of 0.5 followed by ReLU activation function and a 20 unit hidden layer followed by a softmax layer. The maximum length of the input sentence is set to 128. AdamW optimizer has been used to optimize the network with $\epsilon$ = $10^{-8}$, $\beta _1$ = 0.9 and $\beta _2$ = 0.999. The weight decay is set to 0. A $3 \times 10^{-3}$ learning rate has been used. The batch size is set to 8. The meta-network is a three-layer MLP network with an input layer, two hidden layers of 25 hidden nodes, a dropout layer of probability 0.2, and an output layer of size one followed by a sigmoid activation function to bound the value in [0, 1] range. We use a learning rate of $1 \times 10^{-4}$ to learn the meta-network weights. AdamW optimizer with $\epsilon$ = $10^{-8}$, $\beta _1$ = 0.9, $\beta _2$ = 0.999 and weight decay = 0 has been used to learn the data weights. The trade-off parameter $\lambda$ is set to 0.85 for all our experiments because this value performed well in our initial experiments. Our experiments have used the BART-base^48^ model as a paraphrasing model. The maximum text length has been set to 128, and the minimum text length has been set to 65. We utilized the byte-level BPE tokenizer as employed in the RoBERTa model^55^ for our text processing tasks. A batch size of 8 has been used. We use an AdamW optimizer with $\epsilon$ = $10^{-8}$, $\beta _1$ = 0.9 and $\beta _2$ = 0.999. The model has been optimized with a learning rate of $2 \times 10^{-5}$. Linear rate decay with a warm-up ratio of 0.1 and weight decay of 0.01 has been used. The end-to-end framework is run for 10 epochs and further finetuning of the classification model is performed for 20 epochs. We perform experiments on five randomly sampled seeds and report each experiment’s mean and standard deviation for evaluation. The experiments have been performed on an A100 GPU machine.

The results reported in this work are based on the test set of the long COVID dataset^50^. However, for testing with real articles from the internet, we can simply extract the titles and abstracts from the research articles-these succinctly summarize the work-and pass them to the model for classification. To extract metadata such as titles and abstracts from the articles directly, we leverage the open-source library ‘paperscraper’^56,57^.

## Results

Tables 2 show the mean and standard deviation of the Accuracy, F1, Precision, Recall, and AUC of five methods on long COVID article classification over five runs using different random seeds. We have used four methods, namely Vanilla, EDA, Back Translation, and T5 abstractive summarization, as our baselines for comparison. We observe that our method outperforms the baselines on all five evaluation metrics by a large margin, which indicates the efficacy of our method.

From Table 2, the following observations can be made. First, our approach outperforms Vanilla, implying that the generated texts play a crucial role in improving model accuracy, which can be attributed to its diversity and high quality.

**Table 2:** Results on long COVID article classification dataset. The evaluation metrics used are Accuracy, F1 score, Precision, Recall and AUC (reported in percentage). Mean and std refer to the mean and standard deviation of the evaluation metric over five random seeds.

Method	Accuracy		F1		Precision		Recall		AUC	
	Mean	Std	Mean	Std	Mean	Std	Mean	Std	Mean	Std
Vanilla	62.32	1.02	63.97	3.13	60.51	1.44	68.52	8.29	66.84	1.85
EDA	68.26	2.35	69.35	3.99	66.47	3.66	74.11	12.01	74.61	2.09
Back translation	66.38	0.74	65.48	3.28	66.57	3.27	65.58	9.46	71.14	3.08
T5 Abstractive summarization	65.36	3.35	64.92	3.91	64.75	3.3	65.29	5.99	69.02	2.50
Keyword replacement	65.65	6.19	67.64	4.04	64.62	8.65	72.35	6.92	70.80	5.86
Ours	**82.75**	2.47	**80.59**	2.48	**81.99**	3.42	**79.41**	3.83	**85.50**	3.56

Bold indicates the best performance.

Secondly, our approach significantly outperforms EDA, achieving a 14.5% increase in accuracy and an 11.24% improvement in F1 score compared to EDA. Similar gains were observed for AUC, Precision and Recall metrics. These results suggest that employing rule-based techniques like EDA may introduce noise into the original text, leading to suboptimal performance. For example, EDA’s synonym replacement and random deletion operations could inadvertently remove or replace essential medical terminology words that are critical for classification. In contrast, our method generates sentences by applying paraphrasing operations to the input text. We train a paraphrasing model that incorporates feedback from the text classification model, thereby improving its generation based on the classification model’s performance. Consequently, our approach mitigates the introduction of noise into the text, unlike the rule-based techniques employed by EDA. Furthermore, it is noteworthy that EDA generates between 8 and 16 augmentations for each input sentence, while our approach surpasses EDA’s performance with just a single augmentation per input sentence.

Thirdly, our method outperforms the T5 abstractive summarization baseline. The T5 large model is pretrained on non-medical domains and employed for abstractive summarization of input texts to address data deficiency issues. In comparison, our approach achieves a 17.39% higher accuracy and a 15.67% improvement in F1 score compared to the T5 abstractive summarization baseline, highlighting the effectiveness of our domain adaptive paraphrasing-based approach. We observe a similar trend for AUC, Precision and Recall. The T5 model’s pre-training on non-medical domains may lead to summaries that exclude crucial information necessary for long COVID article classification. Furthermore, the T5 model may prioritize compressing the input text to fit the summarization format, potentially resulting in the exclusion of critical information needed for classification. It may also generate hallucinations^58^. An example of generated example is presented in Table 5. Conversely, our paraphrasing model maintains context and meaning, avoiding information loss found in summarization, and uses feedback from the downstream classification model to ensure generated sentences are beneficial for subsequent text classification (Table 5). Therefore, our method outperforms the T5-based summarization baseline.

**Table 3:** Comparison of our method and the rule-based approach on long COVID article classification dataset. The evaluation metrics used are Accuracy, F1 score, and AUC.

Method	Accuracy	F1	AUC
Rule-based approach	69.56	56.25	69.14
Ours	**82.75**	**80.59**	**85.50**

Bold indicates the best performance.

Fourth, our method performs better than the Back translation baseline. In back translation, we use a sequence-to-sequence model based on Opus-MT. Our method achieves an accuracy improvement of 16.37% and an F1 score improvement of 15.11%. A similar trend is noticed for AUC, Precision and Recall metrics too. Back translation may introduce noise into the generated texts. An example of generated example is presented in Table 5. This noise can arise from potential translation errors and information loss, especially when the input text, specific to the medical domain, is translated to another language (French in our case) and back to English using a non-medical pretrained model, which may introduce unwanted words and alter medical terminology in undesirable ways. In contrast, our approach’s feedback mechanism minimizes such risks by encouraging the paraphrasing model to generate text that are more relevant sentences for the long COVID article classification task than the Back translation baseline. Therefore, our domain adaptive paraphrasing-based approach provides a more effective method for generating additional training data for long COVID article classification.

Fifth, our approach outperformed the Keyword Replacement baseline, where augmentations are generated by substituting one long COVID-related keyword for another. One of the reasons is that some instances in the training dataset might imply a connection to long COVID without explicitly mentioning it, making them unaffected by the Keyword Replacement strategy. In contrast, our method, through adaptive data augmentation, captures a deeper understanding of the text’s context, as reflected in its enhanced performance

Sixth, we compared our method to a rule-based strategy that classifies texts based on specific keywords, leveraging the same keyword list from^50^ as the Keyword Replacement baseline. Our results, summarized in Table 3, emphasize the superiority of our approach, which discerns the nuances of content, over traditional keyword-based search engines that mainly rely on a predefined keyword list.

**Table 4:** Results were obtained from testing 100 articles collected from the internet, evaluated using an LSTM model checkpoint previously trained on the long COVID dataset^50^, serving as the backbone for classification. The evaluation metrics used are Accuracy, F1 score, Precision, and Recall (reported in percentage).

Method	Accuracy	F1	Precision	Recall
Vanilla	61	73.1	70.67	75.71
EDA	61	64.86	**87.80**	51.43
Back translation	63	71.32	77.97	65.71
T5 abstractive summarization	58	69.12	71.21	67.14
Keyword replacement	72	82.72	72.83	95.71
Ours	**79**	**86.62**	78.16	**97.14**

Bold indicates the best performance.

Seventh, majority of baseline methods demonstrated a very high standard deviation compared to our approach on Recall metric. This can largely be attributed to the baseline method’s inability to correctly identify articles pertaining to long COVID, often misclassifying them as non-long COVID articles. Our method, on the other hand, utilizes a domain adaptive approach for generating diverse paraphrases, which significantly enhances the model’s generalization capabilities and robustness. This strategy effectively reduces the standard deviation in the Recall metric, leading to more consistent and reliable results. This inference strongly indicates the superiority of our approach in handling the complexity of long COVID literature classification. Further exploration of this observation is undertaken in the Qualitative Analysis Section.

Eighth, to assess the generalizability of our method, we evaluated the previously trained LSTM-based models on 100 recent articles related to COVID-19 collected from bioRxiv^59^. Of these, 70 articles pertained to long COVID, while the remaining 30 were not related to long COVID. We followed the procedure outlined in the ‘Experimental Setup’ section for this experiment and compared the model’s predictions with manual annotations provided by two senior PhD students. The results are summarized in Table 4. Our method outperformed the baseline methods in terms of accuracy, F1 score, and recall. Although EDA achieved a higher precision than ours, it exhibited substantially lower recall, even below the vanilla baseline, indicating a potential bias towards classifying articles as non-long COVID. In contrast, our model not only maintained a balance between precision and recall but also showed enhanced performance on this new test set, demonstrating the generalization ability of our approach.

**Table 5:** Comparison of original and augmented texts generated by our method and baselines. The first augmented text in the baselines is generated using back-translation method, while the second is from abstractive summarization method. Augmented texts in the “Ours” category are generated by our proposed method.

Original	Augmented
Baselines	
Epidemiology and organ specific sequelae of post acute COVID19 A narrative review Long **COVID** a term coined by **COVID** 19 survivors describes persistent or new symptoms in a subset of patients who have recovered from acute illness Globally the population of people infected with SARS CoV 2 continues to expand rapidly necessitating the need for a more thorough understanding of the array of potential sequelae of **COVID** 19 The multisystemic aspects of acute **COVID** 19....	Long **VOCID** narrative journal a term invented by **VOCID** 19 survivors describes persistent or new symptoms in a subset of patients who have recovered from acute disease The global population of people infected with SARS CoV 2 continues to increase rapidly, requiring a deeper understanding of the range of potential sequelae of **VOCID** 19 The multisystemic aspects of **VOCID** 19....
Assessment of tryptophan and kynurenine as prognostic markers in patients with SARS CoV 2 **Immune dysregulation and inflammation** in patients with SARS CoV 2 is associated with a poor clinical outcome We investigated the value of the inflammatory markers tryptophan and kynurenine in predicting the survival outcome of patients with SARS CoV 2 The study included 252 inpatients with a SARS CoV 2 infection ....	The evaluation of tryptophan and kynurenine as prognostic markers in patients with Dysregulation and Immune Inflammation of SARS CoV 2 in patients with Dysregulation and Immune Inflammation of SARS CoV 2 .... included 252 patients with Immune Inflammation of SARS CoV 2 **CoV 2 in patients with Immune Inflammmunation CoV in patients with Immune Inflammmunulation CoV in patients with Immune Inflammmunation CoV in patients with Immune Inflammulence CoV in patients CoV in patients CoV CoV**....
Ours	
COVID 19 still remains a severe global health threat Despite the high speed development of vaccines that efficiently prevent COVID 19 there are still no effective treatments of the disease once people are infected MicroRNAs are powerful regulators of gene expression They are intensely investigated as therapeutic targets up to the clinical stage In addition microRNAs can be detected in the circulation and thus represent promising diagnostic and prognostic biomarkers for **long COVID 19**	COVID 19 still remains a severe global health threat Despite the high speed development of vaccines that efficiently prevent COVID 19, there still are still no effective treatments for the disease once people are infected MicroRNAs are powerful regulators of gene expression They are intensely investigated as therapeutic targets to the clinical stage In addition microRNas can be detected in the circulation and thus represent promising diagnostic and prognostic biomarkers for **long-term COVID syndrome**.
.... This became an **even larger** concern after the COVID 19 **outbreak** left millions of people dead worldwide and generated huge amounts of **infected** or potentially infected wastes The management and disposal of medical wastes during and post COVID 19 represent a major challenge in all countries but this challenge is particularly great for developing countries that do not have robust waste disposal infrastructure The main problems in developing countries include **inefficient treatment procedures, limited capacity of healthcare facilities**, and improper waste disposal procedures.	.... This became a **major** concern after the **COVID infection** left millions of people dead worldwide and generated huge amounts of **contaminated** or potentially infected wastes The management and disposal of medical wastes during and post COVID **was** a major challenge in **developing country (particularly in developing nations)** A review of the current challenges : The main problems in **developing Countries such as lack of adequate healthcare facilities and lack of skilled workers**.

**Figure 3:**
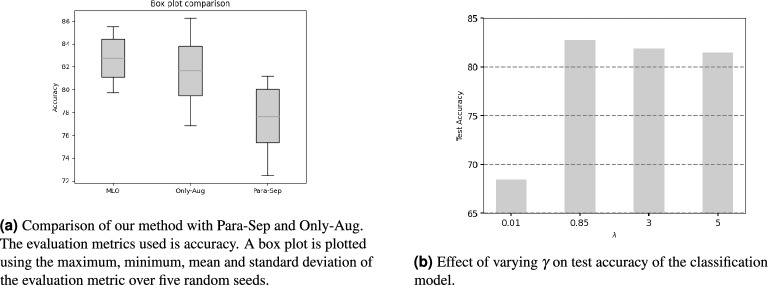
Ablation studies.

### Ablation studies

We perform the following ablation studies to further understand the effectiveness of our method proposed,

**Table 6:** Results on long COVID article classification dataset using the RoBERTa base as the backbone classification model. The evaluation metrics used are Accuracy, F1 score, Precision, Recall and AUC (reported in percentage). Mean and std refer to the mean and standard deviation of the evaluation metric over five random seeds.

Method	Accuracy		F1		Precision		Recall		AUC	
	Mean	Std	Mean	Std	Mean	Std	Mean	Std	Mean	Std
Vanilla	81.59	5.69	81.15	5.01	83.23	8.53	79.71	4.87	88.59	5.21
EDA	80.87	1.75	78.82	3.32	86.37	3.35	73.24	8.23	90.01	0.86
Back translation	79.56	11.74	81.17	8.01	79.46	13.54	84.71	5.31	86.03	10.97
T5 Abstractive summarization	80.43	3.39	80.51	2.63	79.95	5.93	81.76	5.31	87.58	2.78
Ours	**87.10**	1.48	**86.62**	1.45	**88.76**	2.95	**84.71**	2.56	**93.27**	0.93

Bold indicates the best performance.

- Para-Sep: It is an ablation study without the data-reweighting feedback link in our method. The BART-based^48^ paraphrasing and text classification models are trained separately. The paraphrasing model is first trained to produce auxiliary dataset. Then the classification model is trained on its training data and the generated auxiliary data. There is no domain adaptation feedback link. From Fig 3a, our method outperforms Para-Sep baselines by a huge margin. This result highlights the effectiveness of our meta-weight-network-based data reweighting in providing valuable feedback to the paraphrasing model, enabling it to generate more suitable texts in the target domain. While Para-Sep involves separate training of the paraphrasing and text classification models, our approach incorporates a feedback loop where these models influence each other through the data weights. The meta-weight-network parameters are optimized to benefit the downstream text classification task, resulting in superior performance compared to Para-Sep, which lacks such a feedback loop.
- Impact of augmentation dataset (Only-Aug): In this ablation study, we investigate the effect of the generated data on the downstream text classification accuracy during testing. We train the classification model solely on the generated data, excluding the original training dataset. This results in the following optimization problem:
  $$\begin{aligned} \begin{array}{ll} {\text {min}_{W}}\quad &{} L(C^{*}(S^{*}(W)), {\mathscr {D}}^{\text {MLO-val}})\\ \text {s.t.} \quad &{} C^{*}(S^{*}(W)) = L(C, {\mathscr {G}}({\mathscr {D}}^{\text {MLO-tr}},S^{*}(W))) \\ &{} S^{*}(W) = {\text {min}_{S}}\sum \limits _{i=1}^M W(l(S, t_{i}, s_{i})) \cdot l(S, t_{i}, s_{i}) \end{array} \end{aligned}$$
  With reference to Fig 3a, it becomes evident that exclusively training a classification model using generated data could result in compromised performance and high standard deviation. One of the primary reasons for this observation is the inherent advantage of human-curated training data, which is typically characterized by lower noise levels compared to generated data. Consequently, an overemphasis on utilizing generated data might negatively impact the model’s overall performance metrics. Therefore, it is crucial to strike a balance between human-curated and generated data in order to maximize the effectiveness of the classification model.
- Effect of $\gamma$: In this ablation study, we investigate the impact of varying the hyperparameter $\gamma$ on the downstream text classification performance, measured by test accuracy. We examine the effects of $\gamma$ values in the set {0.01, 0.85, 3, 5} on test accuracy. As depicted in Figure 3b, when $\gamma$ is increased from 0.01 to 0.85, the accuracy improves from 68.41% to 82.75%, reflecting an improvement of 14.34%. This suggests that the influence of the generated data grows with increasing $\gamma$. However, further increasing $\gamma$ results in a gradual decline in accuracy. This trend indicates that as $\gamma$ becomes larger, there is an improvement in the model performance owing to an enhancement in the diversity of the training data leading to a better understanding of the task. However by further increasing $\gamma$, the impact of the generated data surpasses that of the training data. Since the original training data is less noisy than the generated data, placing excessive emphasis on the generated data hurt the model’s performance.
- Architecture of downstream classification model: Our data augmentation framework was primarily designed to be agnostic to the choice of downstream model architecture. Initially, we employed a lightweight LSTM model with 15.3M parameters to demonstrate efficacy with less complex architectures. Subsequently, we also experimented with the pre-trained RoBERTa base model^55^, which contains 125M parameters and is a variant of the BERT model^60^. Impressively, our approach consistently outperformed the baselines, including standard RoBERTa fine-tuning, emphasizing the effectiveness of our adaptive data augmentation pipeline. Detailed results with the RoBERTa base model are presented in Table 6. This shows that our method is agnostic to text classifiers and can be leveraged to improve different text classifiers.

### Qualitative analysis

In this subsection, we perform an in-depth qualitative analysis of test examples, focusing on cases where our method correctly classifies instances that all the baseline methods fail to identify. Due to space constraints, we include only a few such instances in Table 7. Intriguingly, all these examples are long COVID-related articles. Upon closer scrutiny, we found that many of these misclassified examples do not contain the term ‘long COVID’ or ‘Sequelae’ (indicating the continuation of the disease). Despite the absence of these terms, our model manages to correctly classify these examples as long COVID articles, while the baselines failed. This can be attributed to the training of our method on diverse training and good quality generated examples, which has enhanced its robustness. Consequently, our model is capable of extracting and leveraging significant features, going beyond superficial markers like ‘long COVID’ or ‘Post-Acute Sequelae of SARS-CoV-2 infection’. It can identify a range of related words and phrases such as ‘long-term consequences of COVID-19’ and ‘post-COVID’, as well as sentences like ‘At six months after COVID-19, critical illness, death, and new disability’, within the broader context of the article, which varies from one article to another. The ability of our method to accurately classify these articles thus demonstrates its effectiveness in discerning and understanding the deeper, more intricate characteristics of long COVID articles, marking a notable improvement over traditional baseline methods.

**Table 7:** Qualitative analysis: This table presents selected examples of texts that our method correctly classified, while all the baseline methods failed to do so. All these instances belong to the ‘long COVID’ class.

“Health related quality of life issues including symptoms in patients with active COVID 19 or post COVID 19 a systematic literature review: This systematic review was performed to identify all relevant health related quality of life HRQoL issues associated with COVID 19. A systematic literature search was undertaken in April 2020. In four teams of three reviewers each all abstracts were independently reviewed for inclusion by two reviewers. Using a pre defined checklist of 93 criteria for each publication data extraction was performed independently by two reviewers and subsequently compared and discussed. If necessary a third reviewer resolved any discrepancies. The search was updated in February 2021 to retrieve new publications on HRQoL issues including issues....”	
“CSF rhinorrhoea post COVID 19 swab - A case report and review of literature: We report the case of a 59 year old male who presented with 2 months of persistent rhinorrhoea from left nostril post a nasal swab done for coryzal symptoms at the peak of the COVID 19 pandemic. Beta 2 transferrin confirmed it to be a CSF leak and imaging showed a left middle cranial fossa encephalocele herniating into the sphenoid sinus as the site of the leak post swab. The leak was treated endoscopically We describe....”	
“Pathogenesis of taste impairment and salivary dysfunction in COVID 19 patients: Coronavirus disease 2019, COVID 19, is a highly transmissible pandemic disease caused by severe acute respiratory syndrome coronavirus 2 SARS CoV 2. The characteristics of the disease include a broad range of symptoms from mild to serious to death with mild pneumonia to acute respiratory distress syndrome and complications in extrapulmonary organs. Taste impairment and salivary dysfunction are common early symptoms in COVID 19 patients. The mouth is a significant entry route for SARS COV 2 similar to the nose and eyes. The cells of the oral epithelium taste buds and minor....”	
“The impact of COVID 19 critical illness on new disability functional outcomes and return to work at 6 months a prospective cohort study There are few reports of new functional impairment following critical illness from COVID 19 We aimed to describe the incidence of death or new disability functional impairment and changes in health related quality of life of patients after COVID 19 critical illness at 6 months ....At six months after COVID 19 critical illness death and new disability was substantial ...”	
“A map of metabolic phenotypes in patients with myalgic encephalomyelitis chronic fatigue syndrome Myalgic encephalomyelitis chronic fatigue syndrome ME CFS is a debilitating disease usually presenting after infection Emerging evidence supports that energy metabolism is affected in .....”	

### Extension to multi-class classification

We aim to extend our method to categorize long COVID articles into more granular sub-classes, facilitating easier access and better organization. This enhancement will streamline database maintenance and improve the efficiency of information retrieval for healthcare professionals. However, this will necessitate a significant investment in time and resources, which we plan to address in our future work.

**Table 8:** Results on BioCreative LitCovid dataset^61^using the LSTM as the backbone classification model. The evaluation metrics used is Accuracy, F1 score, Precision, Recall and AUC (reported in percentage). F1 score, Precision, and Recall are computed using a macro-averaged approach, where each class is treated equally and metrics are averaged across all classes. AUC is calculated with a one-vs-one approach, assessing the classifier’s performance on each pair of classes as binary classification problems, then averaged. Mean and std refer to the mean and standard deviation of the evaluation metric over five random seeds. The dataset categorizes a COVID-19 related research article into seven categories.

Method	Accuracy		F1		Precision		Recall		AUC	
	mean	std	mean	std	mean	std	mean	std	mean	std
Vanilla	53.98	1.21	23.29	0.9	25.09	0.9	23.43	0.57	59.86	2.03
EDA	59.77	1.11	31.32	0.99	33.78	0.49	30.80	1.17	65.10	2.14
Back translation	58.19	2.01	26.36	4.39	27.49	5.7	26.97	3.54	64.50	3.27
T5 Abstractive summarization	60.33	1.66	29.91	2.45	32.77	3.4	30.22	2.02	65.11	1.77
Ours	**66.22**	2.24	**35.72**	1.98	**36.99**	2.09	**35.92**	1.83	**69.6**	0.78

Bold indicates the best performance.

Nevertheless, we perform a preliminary analysis of how our proposed method works in a multi-class classification setup. Hence, we evaluate our method’s efficacy in generating high-quality augmentations for a multi-class classification task. Given the limited availability of annotated long COVID datasets for evaluation, we opted to conduct additional experiments on the BioCreative LitCovid dataset^61^. This dataset is a multi-class COVID-19 article classification dataset, categorizing research articles based on title and abstract into seven distinct categories: ‘Case Report’, ‘Diagnosis’, ‘Epidemic Forecasting’, ‘Mechanism’, ‘Prevention’, ‘Transmission’, and ‘Treatment’. To simulate a low-resource scenario, we selected 667 instances from the entire training dataset. Additionally, our validation and test datasets comprise 333 and 1511 instances, respectively. The class distribution across the training, validation, and test sets is consistent, with Prevention at 49%, Treatment at 16%, Diagnosis at 14%, Case Report at 12%, Mechanism at 5%, and both Epidemic Forecasting and Transmission at 2% each. We conducted experiments with this dataset following the setup in the paper. As shown in Table 8, our method demonstrated performance improvements over baseline approaches, underscoring its capacity to generate informative augmentations, enrich the training dataset, and enhance classifier performance, even in multi-class classification settings.

## Conclusions

In this work, we propose a data-reweighting-based multi-level optimization framework with a meta-weight network for domain-adaptive paraphrasing, specifically designed to generate high-quality additional data for long COVID-related text classification. This framework addresses the prevailing challenge of limited datasets in this domain, ensuring the generation of high-quality additional data, which in turn, enhances the performance of machine learning models. Our framework trains a paraphrasing model and a long COVID article classification model with a feedback mechanism to improve the paraphrasing model based on the performance of the long COVID article classification model. Thus we ensure that the generations from the paraphrasing model are advantageous for the long COVID article classification model. Our framework consists of three stages that are performed in an end-to-end fashion, 1) Training a paraphrasing model, 2) Paraphrasing the training dataset to generate additional dataset and use it to train the long COVID article classification model, 3) Updating the data weights of the paraphrasing model by minimizing the validation loss of the long COVID article classification model. A meta-weight-network is used to learn the data weights distribution of the paraphrasing model. Through extensive experimentation, our approach demonstrates significant improvement over the baselines in addressing data scarcity challenges, underscoring its potential to support the clinical community for long COVID related article/document classification.

In conclusion, this work establishes a solid foundation for future progress and investigations in the realm of machine learning-assisted long COVID research. As we continue our research endeavors, we plan to incorporate diverse data sources, such as electronic health records and social media posts, to bolster the robustness and comprehensiveness of our classification models. We further intend to expand the scope of classification tasks by including more labels and categories, enabling a more comprehensive representation of the intricacies associated with long COVID symptoms and treatment options. In addition, we will evaluate the effects of the evolving terminology related to long COVID on classification performance, ensuring that our models remain current and pertinent as our understanding of the condition advances. We further recognize the value of extracting key information and summaries from papers. Currently, our focus is on classification, but we see potential for advancing to summarization tasks. Building such features is complex, but our classification system lays a foundational groundwork for it. We further plan to club all these functionalities and maintain a long COVID related database. By pursuing these focused future research directions, we aim to significantly contribute to the development of highly effective and efficient tools for long COVID research and clinical practice.

## Data Availability

The dataset analyzed during the current study are available on Huggingface, https://huggingface.co/datasets/llangnickel/long-covid-classification-data and https://ftp.ncbi.nlm.nih.gov/pub/lu/LitCovid/biocreative/.
